# Prevalence of psychosocial findings and their correlation with TMD symptoms in an adult population sample

**DOI:** 10.1186/s40510-024-00538-y

**Published:** 2024-10-14

**Authors:** Giorgio Iodice, Ambra Michelotti, Vincenzo D’Antò, Stefano Martina, Rosa Valletta, Roberto Rongo

**Affiliations:** 1 Department of Neurosciences, Reproductive Sciences and Oral Sciences, School of Orthodontics, University of Naples Federico II, Via Pansini, 5, Naples, 80131 Italy; 2 Department of Medicine, Surgery and Dentistry “Scuola Medica Salernitana”, University of Salerno, Via Al-lende, Baronissi, 84081 Italy

**Keywords:** Population-based, Depression, Anxiety, Temporomandibular disorders, Oral behaviour

## Abstract

**Background and aim:**

Some studies suggested an association between Temporomandibular Disorders (TMD) and psychosocial status, but most of them are focused on samples of patients looking for treatment or present limits of sample representativeness. The aim of the present study was to evaluate the psychosocial status in a large sample of adult population, further than to assess its association to TMD symptoms, oral behaviours, and self-reported facial trauma.

**Results:**

the study sample included 4299 subjects older than 18 years randomly recruited from general population in public spaces during their daily life (1700 Males, 2599 Females mean ± SD age = 40.4 ± 18.1). Psychosocial status and pain-related disability were assessed by means of Patient Health Questionnaire 4 (PHQ-4) and Graded Chronic Pain Scale (GCPS). TMD symptoms were assessed by RDC/TMD and validated screening tools for TMD pain. Oral Behaviours Checklist was used to investigate on oral behaviours. Logistic regression model was used to evaluate the association of the psychosocial status, TMD symptoms, trauma, and oral behaviours. The association was tested using both univariate and multivariate models. The PHQ4 evaluation showed a severe impairment in 4.6% of our sample, moderate in 18.8% and mild in 32.5%. We found a Characteristic Pain Intensity (CPI) level and Interference Score greater that 30 respectively in 36.2% and 22.2% of the study sample. The GCPS status revealed a high disability with severe limitation in 2.5% of the sample, high disability with moderate limitation in 7.0%, low disability high pain intensity in 7.4% and low disability low pain intensity in 37.8%. Anxiety and depression’s levels were significantly associated with gender, TMD pain, coexistence of TMD Pain and sound, and oral behaviours. GCPS status was significantly associated with age, TMD Pain, coexistence of TMD pain and sound, trauma, and oral behaviours.

**Conclusions:**

In the general population, psychosocial impairment is associated to TMD pain, female gender, and report of oral behaviours. Hence, in adults with TMD accompanied by pain, psychosocial status should also be evaluated.

## Introduction

The introduction of Research Diagnostic Criteria for Temporomandibular Disorders (RDC/TMD) in 1992 and later of Diagnostic Criteria (DC/TMD) in 2014 together with the spreading of the biopsychosocial model of pain have led in the recent years to evaluate pain not just as a sensory process, but as always accompanied by cognitive, emotional, and behavioural aspects which influence patients’ reaction and description of pain [1–3].

This specific process related to pain determines coping strategies that may be helpful or harmful in maintaining adequate functioning [3]. Indeed, several psychosocial features have been recognized as risk factors for the development of chronic pain in musculoskeletal disorders and have been identified for chronicity in individuals with TMD [4–6], deeply influencing the patients’ quality of life, increasing health care services utilization and related social costs [7].

Actually, TMD aetiology may involve both pathophysiological and psychosocial factors [3, 8–10]. Stress, fatigue, anxiety, and depression may negatively affect the human psychological status, possibly increasing muscular related TMD [11].

In the last decades the associations between TMD and psychosocial disorders was widely investigated indeed associations were found between: the presence of TMD signs and symptoms and psychosocial disorders as anxiety and depression [8, 12]; the presence of TMD with psychosocial impairment with higher prevalence of muscle disorders than articular disorders [13–15]; the presence of depressive symptoms and TMD pain, independently if articular or muscular [14, 16, 17]. Nevertheless, most part of these studies was realized on samples of seeking treatment adult patients [8, 14, 15, 17, 18]. Only few studies evaluated these associations in the general population [10, 17, 19–22], but some of them did not use validated tools for the evaluation of anxiety and depression or did not used validated and standardized screening questionnaires [8, 23].

Therefore, the aim of the present study was to evaluate the depression and anxiety status in an adult population sample, to assess its association with TMD symptoms, oral behaviours, and self-reported facial trauma. Null hypothesis to test was that psychosocial status and impairment is equally associated to TMD symptoms, pain, and sound.

## Methods

The study sample was randomly recruited among the general population living in the Campania region, Italy. A single operator recorded the data by face-to-face interviews, personally realized in public spaces during their daily life (i.e., supermarkets, cinema, shopping centres, etc.), in order to avoid any bias of age, gender, cultural and/or working influences. Exclusion criteria were subjects under the age of 18, insufficient comprehension of the Italian language, and positive self-report for systemic and/or psychiatric diseases. The subjects fulfilled the questionnaire personally, with the possibility to ask to the operator in case of unclear questions or doubts.

History of the patient was collected using a specific questionnaire recording gender, age, and occurrence of traumatic events in the facial area (i.e. Have you ever had an injury to your face or jaw, with a dichotomous answer “no, yes”).

The psychosocial status was evaluated by means of Graded Chronic Pain Scale (GCPS) and the 4-items Patient Health Questionnaire-4 (PHQ-4) for depression and anxiety. The GCPS is composed of six items assessed on a 10-point numeric rate scale, and one item on the number of disability days due to facial pain. The scoring criteria allow categorizing pain patients into five levels of chronic pain grades (GCPS status: 0, no disability; 1, low disability, low pain intensity; 2, low disability, high pain intensity; 3, high disability, moderately limiting; 4, high disability, severely limiting) [24]. In our study GCPS was submitted to all participants, to evaluate the influence and interference of TMD pain in their daily life. To investigate the degree of depression and anxiety of the sample, the PHQ-4 [25, 26] was used, which present 2 questions on depression and 2 questions on anxiety. The PHQ-4 response options are “not at all”, “several days”, “more than half the days”, and “nearly every day”, scored as 0, 1, 2, and 3, respectively. Therefore, the PHQ-4 Total score ranges from 0 to 12. For the PHQ-4 scale, PHQ-4 Total ≥ 6 was suggested as cut-off point between the normal range and cases of depression or anxiety [25, 27, 28]. PHQ-4 score identifies 4 statuses: PHQ-4 mild (PHQ-4 Total ≥ 3), PHQ-4 moderate (PHQ-4 Total ≥ 6), PHQ-4 severe (PHQ-4 Total ≥ 9) [25–28].

The questions used to investigate on self-reported oral behaviours and TMD symptoms have been deeply described in a previous publication [29]. Questions number 3,4,5,12 and 13 have been selected from the Oral Behaviour Checklist using 5-point scale answers [30]. After collecting the data, oral behaviours checklist output was transformed in a two-way answer. A positive score (i.e., subject with oral behaviours) was given when the answers were either “very often” or “always” for at least one of the behaviours. To investigate on TMJ sounds, questions 15a and 15b of the Research Diagnostic Criteria were used [1] TMD-pain was investigated by means of a validated 3-items screening questionnaire for pain-related TMD [31]. The quantitative evaluation of the answers was scored according to Gonzalez et al.: i.e. for the first question “No pain” (0 points), “From very brief to more than a week, but it does stop” (1 point) and “Continuous” (2 points). All the other questions had only a binary response, where “No” response received 0 points, “Yes” response 1 point. The threshold value for a positive score was 2 (i.e., presence of TMD-pain) [31].

### Sample size calculation

The sample size was calculated conservatively assuming a prevalence of 50% of TMD-pain/TMJ clicking/TMJ crepitus symptoms, a 95% confidence interval and a 1.5% accuracy (3% width). The minimum required sample size was estimated at 4300 subjects. In order to select a representative sample of the population, assuming a response rate of 70%, approximately 6100 subjects will be selected. To avoid any selection bias, we evaluated weighted prevalence of TMD symptoms standardizing for the Italian population.

### Statistical analysis

After data collections, the study sample was split into four age groups (Group 1: >18yrs, ≤ 30yrs; Group 2: >30yrs, ≤ 45yrs; Group 3: >45yrs, ≤ 60yrs; Group 4: >60yrs).

The psychosocial status and frequencies of TMD symptoms were analysed in the relative age groups and in the total sample. Prevalence, with 95% confidence interval, of depression and anxiety were calculated.

Logistic univariate regression model was used to evaluate the association of the psychosocial status described by PHQ-4 Total as dependent variables with TMD symptoms (i.e., TMD-pain, TMJ sound, TMD pain and sound), trauma and oral behaviours as independent variables. Multivariate regression models were used to adjust for age group and gender. Finally, multinomial regression models were used to assess the association between TMD symptoms, anamnestic data, oral behaviours as independent variables and GCPS status or PHQ status as dependent variables. This model was adjusted for age and gender in the multivariate multinomial regression. Results of regression models were reported as odds ratios (OR) and 95% confidence intervals (C.I.). Statistical significance was set at *p* < 0.05. All the statistical procedures were performed with the Statistical Package for the Social Sciences (SPSS 20.0, SPSS Inc., Chicago, Il, USA).

## Results

**Fig. 1:**
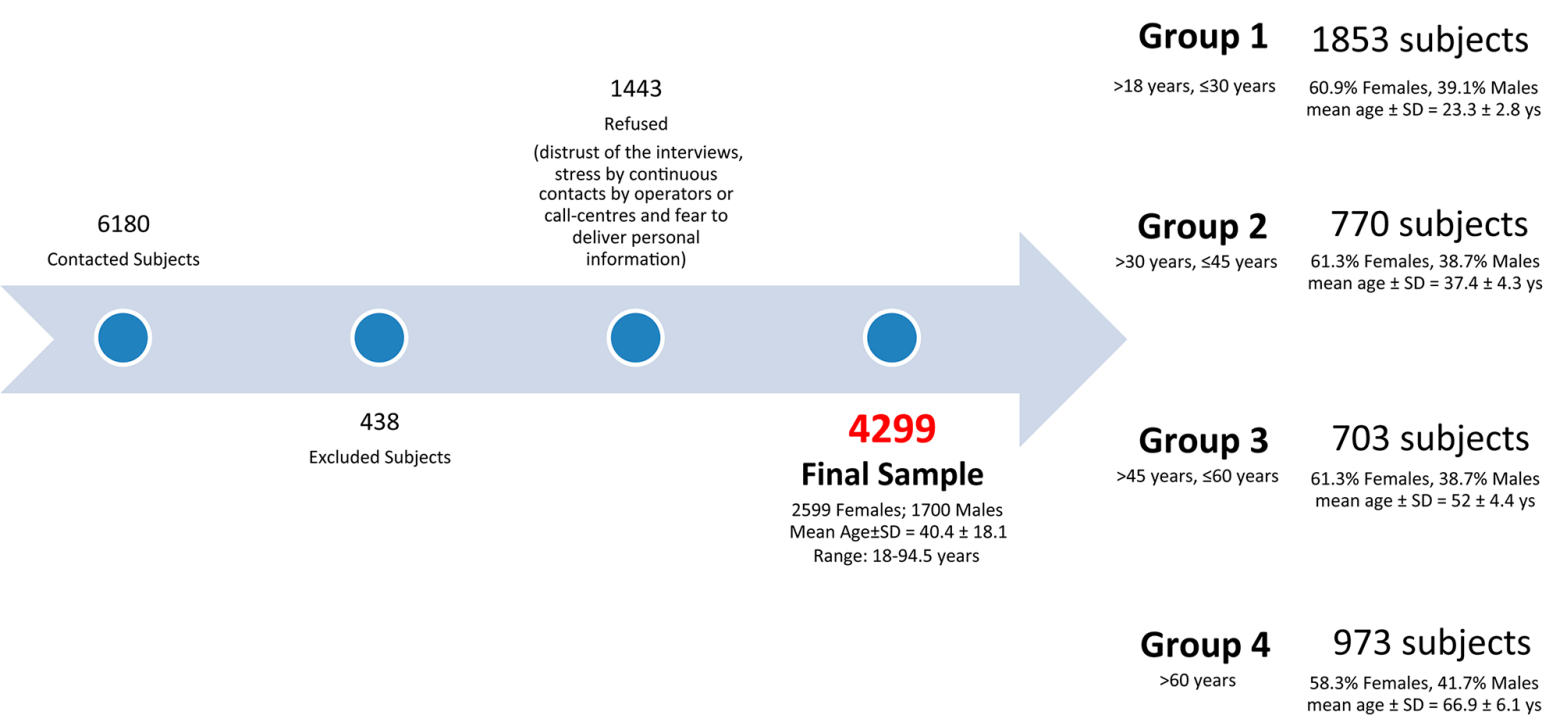
Details about the Sampling Procedure Used for the Study and distribution for age group and gender

Six thousand one hundred eighty subjects were contacted to fill the questionnaire and four hundred thirty-eight subjects were excluded based on the exclusion criteria. One thousand four hundred forty-three subjects refused to be included in the study mainly due to distrust of the interviews and fear to deliver personal information, the final sample included 4299 subjects (60.5% females) ranging from 18 to 94.5 years old (mean ± SD age = 40.4 ± 18.1; Fig. 1).

After splitting into age groups, the study sample accounted for a total of 1853 subjects in group 1 (60.9% females, 39.1% males; mean age ± SD = 23.3 ± 2.8yrs), 770 in group 2 (61.3% females, 38.7% males; mean age ± SD = 37.4 ± 4.3yrs), 703 in group 3 (61.3% females, 38.7% males; mean age ± SD = 52 ± 4.4yrs); and 973 in group 4 (58.3% females, 41.7% males; mean age ± SD = 66.9 ± 6.1yrs).

The prevalence of TMD symptoms, psychosocial status, as well as oral behaviours and trauma are reported in Table 1, as a total and divided for gender and age group. Considering the subjective difficulty in general population to distinguish between TMJ clicking and crepitus, during statistical analysis TMJ clicking and crepitus were merged (i.e. patients with positive report at least at one of the two variables) and evaluated as “TMJ sound” present in 32% of the sample. Furthermore, we separated subjects reporting TMD pain and without TMJ sound (“Pure Pain”) from subjects reporting TMJ sound and not TMD pain (“Pure Sound”). The most common TMD symptom in the sample was TMJ Pure sound (23%; 22.7% males and 23.1% females), followed by TMD pain and sound (8.7%; 7.2% males and 9.7% females) and TMD Pure pain (7.6%; 5.2% males and 9.1% females).

**Table 1:** Prevalence (%) of TMD symptoms and history data in the whole group and split for gender and age groups

	ALL	By Age				Females, by Age					Males, by Age				
		18-30yrs	30.1-45yrs	45.1-60yrs	> 60.1 yrs	Female	18-30yrs	30.1-45yrs	45.1-60yrs	> 60.1 yrs	Male	18-30yrs	30.1-45yrs	45.1-60yrs	> 60.1 yrs
**Psycosocial Status**															
PHQ4 n (%)															
normal	1895 (44.1)	783 (41.3)	381 (20.1)	330 (17.4)	401 (21.2)	962 (37.0)	420 (43.7)	193 (20.1)	164 (17.0)	185 (19.2)	933 (54.9)	363 (38.9)	188 (20.2)	166 (17.8)	216 (23.2)
mild	1396 (32.5)	642 (46.0)	240 (17.2)	182 (13.0)	332 (23.8)	877 (33.7)	401 (45.7)	163 (18.6)	119 (13.6)	194 (22.1)	519 (30.5)	241 (46.4)	77 (14.8)	63 (12.1)	138 (26.6)
moderate	808 (18.8)	326 (40.3)	124 (15.3)	142 (17.6)	216 (26.7)	606 (23.3)	234 (38.6)	95 (15.7)	114 (18.8)	163 (26.9)	202 (11.9)	92 (45.5)	29 (14.4)	28 (13.9)	53 (26.2)
severe	200 (4.6)	99 (49.5)	26 (13.0)	44 (22.0)	31 (15.5)	154 (5.9)	73 (47.4)	20 (13.0)	34 (22.1)	27 (17.5)	46 (2.7)	26 (56.5)	6 (13.0)	10 (21.7)	4 (8.7)
CPI > 30 n (%)	1680 (39.1)	608 (36.2)	299 (17.8)	305 (18.2)	468 (27.9)	1135 (43.7)	423 (37.3)	209 (18.4)	204 (18.0)	299 (26.3)	545 (31.1)	185 (33.9)	90 (16.5)	101 (18.5)	169 (31.0)
Interference Score >30 n (%)	955 (22.2)	334 (35.0)	194 (20.3)	216 (22.6)	211 (22.1)	677 (26.0)	243 (35.9)	142 (21.0)	149 (22.0)	143 (21.1)	278 (16.4)	91 (32.7)	52 (18.7)	67 (24.1)	68 (24.5)
GCP Status n (%)															
no disability	1950 (45.4)	867 (44.5)	383 (19.6)	299 (15.3)	401 (20.6)	1061 (40.8)	475 (44.8)	206 (19.4)	167 (15.7)	213 (20.1)	889 (52.3)	392 (44.1)	177 (19.9)	132 (14.8)	188 (21.1)
low disability/ low pain intensity	1623 (37.8)	725 (44.7)	261 (16.1)	228 (14.0)	409 (25.2)	1011 (38.9)	468 (46.3)	167 (16.5)	144 (14.2)	232 (22.9)	612 (36.0)	257 (42.0)	94 (15.4)	84 (13.7)	177 (28.9)
low disability/ high pain intensity	319 (7.4)	102 (32.0)	59 (18.5)	70 (21.9)	88 (27.6)	219 (8.4)	73 (33.3)	45 (20.5)	44 (20.1)	57 (26.0)	100 (5.9)	29 (29.0)	14 (14.0)	26 (26.0)	31 (31.0)
high disability/ moderately limiting	301 (7.0)	126 (41.9)	51 (16.9)	70 (23.3)	54 (17.9)	225 (8.7)	90 (40.0)	39 (17.3)	50 (22.2)	46 (20.4)	76 (4.5)	36 (47.4)	12 (15.8)	20 (26.3)	8 (10.5)
high disability/ severely limiting	106 (2.5)	30 (28.3)	17 (16.0)	31 (29.2)	28 (26.4)	83 (3.2)	22 (26.5)	14 (16.9)	26 (31.3)	21 (25.3)	23 (1.4)	8 (34.8)	3 (13.0)	5 (21.7)	7 (30.4)
**TMD symptoms**															
TMD Pure Pain, n (%)	326 (7.6)	128 (39.3)	59 (18.1)	76 (23.3)	63 (19.3)	237 (9.1)	91 (38.4)	40 (16.9)	56 (23.6)	50 (21.1)	89 (5.2)	37 (41.5)	19 (21.3)	20 (22.5)	13 (14.6)
TMJ Pure Sound, n (%)	987 (23.0)	405 (41.0)	171 (17.3)	155 (15.7)	256 (25.9)	601 (23.1)	255 (42.4)	101 (16.8)	99 (16.5)	146 (24.3)	386 (22.7)	150 (38.9)	70 (18.1)	56 (14.5)	110 (28.5)
TMD Pain & Sound, n (%)	375 (8.7)	175 (46.6)	68 (18.1)	72 (19.2)	60 (16.0)	253 (9.7)	117 (46.2)	46 (18.2)	45 (17.8)	45 (17.8)	122 (7.2)	58 (47.5)	22 (18.0)	27 (22.1)	15 (12.3)
**Oral Behaviours**	1250 (29.1)	741 (59.3)	197 (15.8)	168 (13.4)	144 (11.5)	815 (31.4)	473 (58.0)	132 (16.2)	122 (15.0)	88 (10.8)	435 (25.6)	268 (61.6)	65 (14.9)	46 (10.6)	56 (12.9)
**Trauma**	310 (7.2)	140 (45.2)	55 (17.7)	69 (22.3)	46 (14.8)	128 (4.9)	55 (43.0)	21 (16.4)	31 (24.2)	21 (16.4)	182 (10.7)	85 (46.7)	34 (18.7)	38 (20.9	25 (13.7)
**Continuous Variables**															
	**Mean**	**Interquartile Range**													
CPI	16.67	0-36.67													
Disability Score	0	0–20													

The total score of the PHQ-4 revealed a prevalence of 23.4% (29.2% females, 14.6% males) cases of depression or anxiety. According to GCPS, 39% of subjects (43.7% females; 32.1% males) reported high pain-related impairment and 22.2% (26% female; 16.4% males) reported a severe interference of pain in daily life. A mean of 20.5 ± 21.9 (from 0 to 90) and 13.3 ± 22.2 (from 0 to 100) was found for the CPI and Disability score values respectively.

Oral behaviours evaluation revealed that 29.1% of the subjects reported at least one oral behaviour. Thirty-five per cent of the sample investigated used chewing gum “often”, “very often” or “always”, making it the most common oral behaviours. Teeth contact was present in 21.7% of the sample, whereas clenching and grinding in 16.3%. Furthermore, 7.2% of subjects reported a trauma to the face and/or jaw during their life.

In Table 2 univariate and multivariate logistic regression model is reported using PHQ-4 Total as dependent variable, adjusted for age and gender, and TMD symptoms, trauma, and oral behaviours as independent variables. PHQ-4 was significantly associated with female gender (*p* < 0.001; OR = 2.4), Pure pain (*p* < 0.001; OR = 1.8), pain and sound (*p* < 0.001; OR = 2.8), oral behaviours (*p* < 0.001; OR = 2.1), CPI (*p* < 0.001; OR = 2.8) and disability score (*p* < 0.001; OR = 3.0), all the associations were confirmed in the multivariate analysis corrected by gender and age. Table 2 showed the OR of the multinomial logistic analysis for the different degrees of PHQ-4, adjusted, and not adjusted for gender and age. Except for TMJ Pure sound, all the independent variables presented a significant association with the PHQ score, and the OR is higher when PHQ-4 is more severe. Table 3 showed the OR of the multinomial logistic analysis for the different statuses of GCPS as dependent variable, adjusted and not adjusted for age and gender, and TMD symptoms, trauma, and oral behaviours as independent variables. In the different GCPS statuses there was a significant association with different group age, in general younger people are presented lower scores of GCPS. Furthermore, GCPS statuses were associated with female gender, Pure pain, Pain and sound and Oral behaviours, and in general these associations were stronger for more severe status of GCPS.

**Table 2:** Results of univariate and multiple regression analysis with PQH-4 as the dependent variable and gender, age, oral behaviors, trauma and as independent variables

	PHQ-4 TOTAL ≥ 6						PHQ-4 Mild						PHQ-4 Moderate						PHQ-4 Severe					
	Univariate			Multivariate			Univariate			Multivariate			Univariate			Multivariate			Univariate			Multivariate		
	OR	CI	*p*	OR	CI	*p*	OR	CI	*p*	OR	CI	*p*	OR	CI	*p*	OR	CI	*p*	OR	CI	*p*	OR	CI	*p*
**Age**																								
30	0.88	0.74–1.06	0.185				0.99	0.83–1.18	0.91				**0.77**	**0.63–0.95**	**0.016**				**1.64**	**1.07–2.49**	**0.022**			
30/45	**0.72**	**0.57–0.90**	**0.004**				**0.76**	**0.61–0.95**	**0.014**				**0.60**	**0.46–0.78**	**< 0.001**				0.88	0.51–1.51	0.065			
45/60	1.08	0.86–1.34	0.50				**0.67**	**0.53–0.84**	**0.001**				0.80	0.62–1.03	0.087				**1.72**	**1.08–2.79**	**0.027**			
(60)																								
**Gender**																								
(Male)																								
Female	**2.42**	**2.06–2.84**	**< 0.001**				**1.64**	**1.42–1.88**	**< 0.001**				**2.9**	**2.42–3.49**	**< 0.001**				**3.2**	**2.30–4.57**	**< 0.001**			
**TMD Pure Pain**																								
(No)																								
Yes	**1.77**	**1.39–2.25**	**< 0.001**	1.09	0.82–1.44	0.55	**1.46**	**1.11–1.92**	**0.008**	0.99	0.72–1.35	0.95	**2**	**1.49–2.7**	**< 0.001**	1.04	0.74–1.47	0.081	**2.55**	**1.61–4.02**	**< 0.001**	1.27	0.74–2.17	0.38
**TMJ Pure Sound**																								
(No)																								
Yes	1.09	0.93–1.29	0.28	**1.24**	**1.03–1.49**	**0.020**	0.95	0.80–1.12	0.54	0.97	0.82–1.16	0.789	1.1	0.92–1.35	0.28	**1.24**	**1.00-1.53**	**0.046**	0.92	0.65–1.31	0.64	**1.20**	**0.81–1.78**	**0.36**
**TMD Pain & Sound**																								
(No)																								
Yes	**2.80**	**2.25–3.48**	**< 0.001**	**1.63**	**1.26–2.12**	**< 0.001**	**1.36**	**1.03–1.80**	**0.03**	0.96	0.70–1.30	0.78	**2.88**	**2.19–3.80**	**< 0.001**	**1.48**	**1.07–2.05**	**< 0.001**	**4.71**	**3.20–6.94**	**< 0.001**	**2.06**	**1.28–3.31**	**0.003**
**Trauma**																								
(No)																								
Yes	1.28	0.98–1.65	0.064	1.22	0.92–1.62	0.174	**0.62**	**0.47–0.83**	**0.001**	**0.64**	**0.47–0.87**	**0.004**	**0.97**	**0.71–1.32**	**0.039**	0.94	0.67–1.31	0.71	1.49	0.093-2.37	0.093	1.29	0.77–2.12	0.32
**Oral Behavior**																								
(No)																								
Yes	**2.09**	**1.80–2.43**	**< 0.001**	**1.98**	**1.68–2.32**	**< 0.001**	**1.44**	**1.23–1.69**	**< 0.001**	**1.37**	**1.16–1.62**	**< 0.001**	**2.27**	**1.90–2.71**	**< 0.001**	**2.19**	**1.80–2.66**	**< 0.001**	**3.41**	**2.53–4.59**	**< 0.001**	**2.80**	**2.04–3.85**	**< 0.001**
**CPI > 30**																								
(No)																								
Yes	**2.79**	**2.41–3.22**	**< 0.001**	**1.78**	**1.48–2.15**	**< 0.001**	**1.76**	**1.52–2.04**	**< 0.001**	**1.5**	**1.24–1.80**	**< 0.001**	**3.44**	**2.89–4.08**	**< 0.001**	**2.03**	**1.62–2.53**	**< 0.001**	**4.31**	**3.18–5.84**	**< 0.001**	**2.16**	**1.45–3.22**	**< 0.001**
**Disability Score > 30**																								
(No)																								
Yes	**2.99**	**2.56–3.49**	**< 0.001**	**1.77**	**1.44–2.17**	**< 0.001**	**1.84**	**1.53–2.21**	**< 0.001**	1.43	1.14–1.81	0.79	**3.76**	**3.09–4.57**	**< 0.001**	**2.02**	**1.57–2.61**	**< 0.001**	**5.03**	**3.69–6.85**	**< 0.001**	**2.24**	**1.48–3.39**	**< 0.001**

Bold format indicates statistically significant associations (*p* < 0.05)

**Table 3:** Results of multiple regression analysis with GCPS as the dependent variable and gender, age, oral behaviors, trauma and as independent variables, in the multivariate model was adjusted for gender and age

	GCPS 1 - low disability/ low pain intensity						GCPS 2 - low disability/ high pain intensity						GCPS 3 - high disability/ moderately limiting						GCPS 4 - high disability/ severely limiting					
	Univariate			Multivariate			Univariate			Multivariate			Univariate			Multivariate			Univariate			Multivariate		
	OR	CI	*p*	OR	CI	*p*	OR	CI	*p*	OR	CI	*p*	OR	CI	*p*	OR	CI	*p*	OR	CI	*p*	OR	CI	*p*
**Age**																								
30	**0.82**	**0.69–0.97**	**0.022**				**0.53**	**0.39–0.73**	**< 0.001**				1.08	0.77–1.52	0.66				**0.49**	**0.29–0.84**	**0.009**			
30/45	**0.66**	**0.54–0.82**	**< 0.001**				0.70	0.49-1.00	0.053				0.99	0.66–1.49	0.96				0.64	0.34–1.18	0.151			
45/60	**0.74**	**0.60–0.93**	**0.010**				1.07	0.07–1.51	0.71				**1.74**	**1.18–2.55**	**0.005**				1.48	0.87–2.53	0.146			
(60)																								
**Gender**																								
(Male)																								
Female	**1.38**	**1.21–1.58**	**< 0.001**				1.83	1.42–2.36	**< 0.001**				**2.48**	**1.88–3.26**	**< 0.001**				**3.02**	**1.89–4.84**	**< 0.001**			
**TMD Pure Pain**																								
(No)																								
Yes	**26.18**	**12.82–53.43**	**< 0.001**	**32.54**	**15.89–66.60**	**< 0.001**	**38.84**	**18.09–83.37**	**< 0.001**	**61.11**	**28.06-133.07**	**< 0.001**	**81.99**	**39.06-172.11**	**< 0.001**	**143.99**	**67.14–308.80**	**< 0.001**	**147.12**	**66.25-326.69**	**< 0.001**	**555.26**	**213.70-1442.72**	**< 0.001**
**TMJ Pure Sound**																								
(No)																								
Yes	1.06	0.09–1.24	0.45	1.39	1.18–1.63	**< 0.001**	1.23	0.94–1.61	0.12	**2.10**	**1.56–2.83**	**< 0.001**	**0.66**	**0.48–0.92**	**0.013**	**1.69**	**1.17–2.45**	**0.005**	**0.39**	**0.20–0.73**	**0.003**	**2.64**	**1.15–6.07**	**0.022**
**TMD Pain & Sound**																								
(No)																								
Yes	**60.31**	**22.34-162.82**	**< 0.001**	**71.68**	**26.49-193.97**	**< 0.001**	**117.27**	**42.34-325.29**	**< 0.001**	**172.41**	**61.51-483.31**	**< 0.001**	**197.78**	**71.88–544.20**	**< 0.001**	**321.10**	**114.77-898.41**	**< 0.001**	**332.06**	**115.64-953.48**	**< 0.001**	**1212.25**	**372.53-3944.76**	**< 0.001**
**Trauma**																								
(No)																								
Yes	**1.59**	**1.21–2.09**	**0.001**	**1.43**	**1.06–1.92**	**0.017**	**1.82**	**1.17–2.82**	**0.007**	1.51	0.95–2.43	0.084	**3.15**	**2.15–4.61**	**< 0.001**	**2.62**	**1.68–4.08**	**< 0.001**	**3.11**	**1.74–5.58**	**< 0.001**	**2.54**	**1.32–4.91**	**0.005**
**Oral Behavior**																								
(No)																								
Yes	**1.44**	**1.24–1.67**	**< 0.001**	**1.36**	**1.16–1.59**	**< 0.001**	**1.77**	**1.38–2.28**	**< 0.001**	**1.78**	**1.36–2.33**	**< 0.001**	**1.84**	**1.42–2.38**	**< 0.001**	**1.49**	**1.11–1.99**	**0.008**	**2.32**	**1.56–3.46**	**< 0.001**	**2.01**	**1.28–3.17**	**0.002**

Bold format indicates statistically significant associations (*p* < 0.05)

## Discussion

We validated the external validity of our sample as representative of the general Italian population [29]. Over the last decades international literature has confirmed that TMD aetiology may involve both pathophysiological and psychosocial factors [9, 10, 12], suggesting a greater implication of the psychosocial factor, in comparison to other physical ones [10, 20, 32–34]. The psychosocial evaluation of the TMD patient has been standardized in RDC/TMD by means of useful assessment tools. However, very few studies investigated on the prevalence of GCPS and the levels of depression and somatization in patients with different TMD diagnoses [22, 35–37], analysing the correlations. Furthermore, most part of the studies on the topic used samples of patients asking for orthodontic or TMD treatment or was very limited to a specific age range. These represent severe limits to the external validity of the findings reported, deeply influencing the possibility to export the results to the general population [10, 19–22, 38]. Moreover, very few of them used standardized and validated screening tools for TMD symptoms as well as for psychosocial status [10, 39]. Conversely, accordingly to the suggestions of a systematic review, in this study we use standardized and reliable screening tools, which can help to prevent more idiosyncratic and unstructured assessments of psychological comorbidity [3]. As far as we know, the present study is the first one realised by standardized and validated screening tools on a large sample of general population.

Our main finding confirmed that TMD symptoms as well as psychosocial diagnostic subgroups are common also in non-patient populations. Both psychosocial investigating tools (i.e. GCPS and PHQ-4) were found to be associated to TMD Pure pain and oral behaviours, whereas no association was found with TMJ Pure sound. Based on reciprocal interactions between the different TMD symptoms, and considering them as confounding factors, in the statistical analysis of the present study we evaluated both the single symptoms present exclusively (i.e. pain but not sound and vice versa) and in combination (i.e. pain in conjunction with sound simultaneously). In the present investigation the most common TMD symptom was TMJ Pure sound, present as a single symptom in 23% of the sample and in 8.7% as a simultaneous combination to TMD pain. The prevalence of TMJ sound was consistent with previous findings, ranging from 18% to 35% [40–43]. This finding is also consistent with a systematic review on the topic, which reported disc displacement with reduction as the commonest diagnosis in community sample studies, whereas pain is the commonest diagnosis in TMD patient populations [22]. The prevalence of TMD-pain was 7.6% as a single symptom and 8.7% in combination to TMJ sound. These data agree with international surveys reporting prevalence ranging from 13 to 21% [41, 44], but they are divergent to an investigation on an Italian population sample reporting a prevalence of only 5.1% [42]. Possible reasons for the discrepancy could be ascribed to the different methodologies in TMD-pain investigation tool as well as the duration of their investigation.

Even though PHQ-4 can be split into two different tools investigating on anxiety and depression independently (i.e. GAD-2 and PHQ-2), as suggested by the authors we used it as a single score based on all 4 items [2, 23, 27]. The evaluation of the psychosocial status revealed a prevalence of 23.4% of moderate or severe distress status [23, 27]. Most part of the subjects reported a “normal” or “mild” distress status for both genders, even though females presented a significant higher prevalence of “moderate” and “severe” distress compared to males (OR 2.4; *p* < 0.001). Logistic regression model reported significant association between PHQ-4 status and TMD pain and Pain and Sound. Very interestingly, there is a gradient of association, with a strength becoming stronger and stronger from PHQ-4 mild status to severe one for all the significant variables. No significant association was found between PHQ-4 status and TMJ Pure sound (Table 2). This trend remarks that as greater is the pain reported by the subjects as greater is their mental impairment, anxiety, and depression. Interestingly, about almost one third of the subjects reporting TMJ sound presented also TMD pain concurrently. This confirms the importance to describe the prevalence of muscle and joint disorders independently and not as a whole entity in the general population, in order to avoid bias of confounding influences between the different TMD symptoms.

According to GCPS, 39% of our sample reported high pain-related impairment and 22.2% severe interference of pain in daily life. Similarly, to PHQ-4, most part of the subjects reported GCPS grade 0 and 1 for both genders, even though females presented a significant higher prevalence of chronic pain impairment compared to males. Logistic regression model reported significant association between GCPS grade and TMD Pure pain, Pain and sound, oral behaviours, and traumas. Also, for GCPS the strength of association becomes stronger and stronger going from score 0 to 4 for all the significant variables. No significant association was found between GCPS grade and TMJ Pure sound (Table 2).

Logistic regression model also reported a significant association between oral behaviours and both PHQ-4 status and GCPS grade. These results thus clearly show an association between TMD and stress, consistently with previous studies [19, 45, 46]. Indeed, our data indicate that oral behaviours and awake bruxism are associated with mood states, such as anxiety. Even though we have to remark that our findings, as most part of the studies investigating this topic [47, 48] are related to self-reported oral behaviours, similar results have been reported using quantitative analysis as electromyography [45].

Very interestingly all the associations we found presented a biological gradient, indeed as greater was the reported pain, Pain and sound or oral behaviour presence as stronger was the association found with both PHQ-4 and GCPS. The presence of this biological gradient, reported among the Bradford Hill’s criteria for causality, contributes to strength the association between these TMD symptoms and the psychosocial impairment [49].

Strong association was finally found between trauma and pain-related impairment (GCPS scores), possibly suggesting the contribution of general pain to TMD Pure pain in the psychosocial impairment.

Our findings confirm the results of previous studies [10, 19–21, 50], reporting correlations between anxiety and depression and TMD symptoms, as well as parafunctions habits. However, compared to these previous publications, our study presents the strength of a very large sample representative of the general population, as well as the use of standardized and validated tools for both TMD and psychosocial assessment. On the other side, we must remark that our study assessed just TMD self-reported symptoms, lacking a clinical visit to investigate on TMD signs. However, a previous epidemiologic study compared TMJ signs and symptoms, reporting high accuracy of self-reported TMJ clicking, with a sensitivity of 0.47 and specificity of 0.99 [51].

Hence the analysis of our data lead to refuse the null hypothesis that psychosocial status and impairment is equally associated to TMD symptoms, pain, and sound. These findings can support, instead, the hypothesis that somatization and somatosensitive amplification are highly present in subject with high score of psychosocial impairment (PHQ-4 and GCPS).

The lack of a clinical examination represents a limit to the present study. However, the use of standardized and validated tools specifically introduced for large sample investigations may counterbalance this lack, strengthening the power of the findings. Furthermore, the large sample size and the community-based design support the external validity of our results, leading to consider them as representative of Italian general population.

## Conclusions

The findings of our study remark that TMD symptoms as well as psychosocial diagnostic subgroups are common also in non-patient populations. The presence TMJ sound was found not to be associated to psychosocial depression and somatization. Conversely, higher levels of TMD pain were found to be associated to greater psychological impairment. Furthermore, significant association was also found between depression and somatization, female gender, and report of oral behaviours. Hence, we can conclude that, evaluating subjects reporting TMD symptoms, emotional status should be considered during diagnosis. The presence of TMD pain and lasting oral behaviours need to be addressed and stopped, in order to avoid potential psychosocial impairment, anxiety and depression.

## Data Availability

The data presented in this study are available on request from the corresponding author.
